# Albumin infusion in hospitalized patients with acute heart failure: a retrospective cohort study

**DOI:** 10.1186/s12872-022-02797-1

**Published:** 2022-08-06

**Authors:** Lei Wang, Yun-Tao Zhao

**Affiliations:** Department of Cardiology, Aerospace Center Hospital, 15 Yuquan Road, Haidian District, Beijing, 100049 People’s Republic of China

**Keywords:** Acute heart failure, Albumin infusion, Mortality

## Abstract

**Background:**

Heart failure is frequently associated with hypoalbuminaemia and poor prognosis. Acute heart failure (AHF) patients are commonly treated with intravenous albumin to improve osmotic pressure and haemodynamics. However, the effects of exogenous albumin supplementation on the fatality rate of AHF patients have not yet been demonstrated. Therefore, the present study strived to examine the impacts of albumin injections on the mortality rate of patients with AHF.

**Methods:**

This retrospective cohort study evaluated the clinical outcomes of all consecutive hospitalized patients. Data were collected from medical records. The primary end-point was a composite of intubation, emergency renal replacement, or mortality in a time-to-event analysis. An inverse probability-weighted multivariable Cox model was used to compare outcomes between patients who were treated with albumin and those who were not based on the propensity score.

**Results:**

Among the 1420 consecutive patients hospitalized in our hospital with acute decompensated heart failure between 1 January 2017 and 27 February 2021, 382 were excluded, 337 (32.5%) were administered albumin (median treatment dose of 29.0 g), and 701 (67.5%) were not. The albumin exposure varied by body mass index, age group, previous diagnoses, clinical signs and symptoms, laboratory tests, and use of other drugs in the unmatched sample. The patients receiving albumin exhibited a lower serum albumin level at baseline in contrast with those who were not treated with albumin (median, 37.3 g/L vs. 31.7 g/L, respectively). Overall, primary end-point events occurred in 357 patients (34.4%) (79 died without being intubated or during an emergency renal replacement therapy, 118 were intubated and 160 had an emergency renal replacement therapy). In the inverse probability weighted multivariable analysis based on the propensity score, albumin use was not significantly associated with the composite primary end-point (hazard ratio, 1.05; 95% confidence interval, 0.75–1.47).

**Conclusion:**

In this observational study of AHF patients hospitalized in our hospital, the administration of albumin did not show a relationship with either a greatly reduced or aggregated risk of the composite end-point of intubation, emergency renal replacement therapy, or death. Therefore, randomized controlled trials of albumin administration are needed for patients with AHF.

**Supplementary Information:**

The online version contains supplementary material available at 10.1186/s12872-022-02797-1.

## Take home message

Heart failure is frequently associated with hypoalbuminaemia and poor prognosis.

The administration of albumin did not show a relationship with either a greatly reduced or aggregated risk of the composite end-point of intubation, emergency renal replacement therapy, or death.

## Background

Heart failure is frequently associated with hypoalbuminaemia and poor prognosis [1, 2]. Albumin administration is a common intervention for patients with acute heart failure (AHF) to improve osmotic pressure and haemodynamics [3], enhance the diuretic effects [4, 5] and relieve oedema and serous effusion. Given the available methods to manage hypoalbuminaemia, interventional studies (such as exogenous albumin supplementation or nutritional status improvement of patients with AHF) are warranted [6, 7].

The human serum albumin (HSA) is the most bountiful protein in the plasma and the primary protein to maintain osmotic pressure. HSA is widely administered as the clinical treatment for hypovolaemic, surgical blood loss, bleeding, shock, extracorporeal circulation, acute respiratory distress syndrome, burns, haemodialysis, acute liver failure, trauma, chronic liver disease, nutritional support, resuscitation, hypoproteinaemia, and other diseases [8].

However, clinical trials of albumin use have shown conflicting results. The use of 4–5% albumin in severely ill patients has not consistently decreased mortality compared with the use of normal saline. A retrospective cohort study showed that 5% albumin was associated with decreased mortality during large-volume resuscitation [9]. A multicentre, randomized, double-blind trial of fluid resuscitation using 4% albumin or normal saline in patients admitted to the intensive care unit showed similar mortality at 28 days [10].

Among patients with serious disease conditions and sepsis, 20% albumin has been found superior to 4–5% albumin but has not consistently decreased the mortality rate as compared with the use of normal saline. An open-label randomized controlled trial indicated that the use of 20% albumin during low-volume resuscitation reduces the need for resuscitation fluid and fluid accumulation in contrast with the use of 4–5% albumin [11]. Treatment with 20% albumin also improved organ function in critically ill patients with hypoalbuminaemia [12]. When contrasted with normal saline, albumin administration did not adversely affect renal or other organ functioning and could reduce the risk of mortality; the adjusted odds ratio for mortality in patients administered albumin vs saline was 0.71 (95% confidence interval [CI] 0.52–0.97; *p* = 0.03) [13]. Furthermore, a meta-analysis reported a lower mortality rate in patients who suffered from sepsis and received resuscitation with a solution containing albumin than in patients who received other liquid resuscitating regimens [14]. However, in a randomized, multicentre, open-labeled trial of 1818 patients with severe sepsis, 20% albumin did not improve at the 28- and 90-day survival compared with crystal alone [15].

Although albumin replacement therapy has been widely used clinically for patients with heart failure, its role in improving the prognosis remains unknown, and the effects of exogenous albumin supplementation on the mortality of patients with heart failure have not been demonstrated.

This retrospective cohort study was conducted to evaluate whether infusing 20% human albumin solution reduces the incidence of death among hospitalized patients with acute decompensated heart failure (ADHF) compared to standard care.

## Methods

### Patient selection

This is a retrospective cohort study that evaluated the clinical outcomes of all consecutive hospitalized patients. 1420 patients diagnosed with ADHF and admitted to the hospital between 1 January 2017 and 27 February 2021 were retrospectively recruited in accordance with the European Society of Cardiology guidelines [16].

Patients who met the following criteria were excluded: pregnant women, patients with dementia and psychosis, patients aged < 18 years, patients on intubation and undergoing emergency renal replacement therapy or surgery, and patients who died before the study baseline and were discharged after an inpatient admission within 24 h.

### Data sources

The electronic medical records of the Electronic Information System were reviewed to retrieve demographic, clinical, and laboratory data. The obtained data were reviewed and cross-checked by a team of experienced cardiologists. Each record was independently reviewed by two clinicians.

The following study variables were collected from each patient: clinical signs and symptoms, laboratory findings, demographic variables, imaging results, medical history, and treatment. Demographic variables included sex, age, height, and weight. Medical history included diabetes, hypertension, coronary artery disease, previous heart failure, atrial fibrillation, previous renal dysfunction, cerebral infarction, cancer, and cirrhosis. Clinical signs and symptoms included the following categorical and continuous variables: New York Heart Association functional class, orthopnoea, rales (> 1/2 lung fields), systolic blood pressure, paroxysmal nocturnal dyspnoea, diastolic blood pressure, heart rate, jugular venous distention, and peripheral oedema. Imaging results included left ventricular ejection fraction (LVEF) obtained using 2D transthoracic echocardiography. The following laboratory findings were also reviewed: B-type natriuretic peptide, C-reactive protein, troponin I, creatinine, haemoglobin, alanine aminotransferase, serum sodium, blood urea nitrogen, albumin, serum potassium, uric acid, and glucose. The baseline values of these tests were recorded, with the first value within 2 days of admission being used. Patients were treated with aldosterone antagonists, beta-blockers, loop diuretics, angiotensin-converting enzyme inhibitors/angiotensin receptor blockers (ACE-Is/ARBs), anticoagulants, aspirin, ADP-P2Y12 antagonists, vasopressors, recombinant human brain natriuretic peptide (rh-BNP) and non-invasive positive pressure ventilation (NPPV). The Supplementary Appendix provides more information on these factors (Additional file 1: Table S1).

### Study baseline

Patients were categorized as having been administered with albumin if they received it at baseline or in the follow-up procedure prior to intubation, emergency renal replacement therapy, or death. The baseline set for this study was 24 h before the albumin administration.

### Albumin infusion

Albumin concentration is 20%. The time duration and doses of albumin infusion were recorded.

### Study end-points

The primary end-points were the duration between the baseline and intubation, emergency renal replacement therapy, or death. In patients who died following intubation or during an emergency renal replacement, the duration of the primary end-point was identified as the period of intubation or emergency renal replacement. The study team reviewed all end-points in detail.

### Sample size

The retrospective nature of this study predetermined the sample size.

### Missing data

Before data analysis, predictor variables were assessed for missing values. Among these, the proportion of missing data was 0.1–8.6%. To include these data from analyses, multiple imputations were performed on missing data through chained equations, using the mice R package, in which predictive mean matching was embedded with the patients, with k = 5 as the default setting. Baseline clinical characteristics at pre-and post-imputation are presented in Additional file 1: Table S2.

### Statistical analysis

For categorical and continuous variables, data were expressed as frequencies (percentages) and means (standard deviations) or medians (interquartile ranges [IQRs]), respectively. The means for continuous variables were compared utilizing *t*-tests in data with normal distribution; otherwise, the Mann–Whitney U test was used. To compare the proportions for categorical variables, the chi-square test was adopted. Fisher’s exact probability test was adopted in the case of limited data. The α = 0.05 and *p* < 0.05 (two-tailed) were considered as significant levels of the statistical analyses.

Cox proportional hazard regression models were employed to evaluate the relationship between the use of albumin and end-point. Demographic characteristics, laboratory tests, clinical parameters, and prescriptions were all included in the multivariable Cox regression model. We selected these confounders based on their relationships with outcomes of interest or changes in effect estimate of > 10%.

Furthermore, propensity-score approaches were utilized to minimize the impact of confounding factors so as to compensate for the non-randomized treatment delivery of albumin [17]. Subsequently, associations between the use of albumin and the end-point were evaluated utilizing multivariable Cox regression models based on two propensity-score methods. The nearest-neighbour approach was employed to generate a matching control sample utilizing propensity-score matching analysis. A logistic regression model of albumin administration was fitted and the predicted likelihood of albumin administration was computed by virtue of regression on other baseline covariates. This study utilized an entry survey to guarantee that all selected variables of patients who were confirmed to have been administered albumin were recruited as covariates in the model to minimize potential confounding variables through an indication. The treatment group were matched in a 1:1 ratio to the control group based on the propensity score without a caliper width. Predicted probabilities were utilized in the IPW analysis to compute the stabilized IPW in the time-to-event analysis. Cox models and Kaplan–Meier curves using IPW analysis were reported.

### Sensitivity analysis

Sensitivity analyses were performed, with propensity score matching different variables with a standard caliper width of 0.2 and without a caliper width. Sub-group analysis were employed to evaluate the relationship between the use of albumin and end-point.

### Statistical analysis software

Data were analyzed by the statistical package R (The R Foundation; http://www.r-project.org; version 4.0.5).

## Results

### Cohort characteristics

**Fig. 1:**
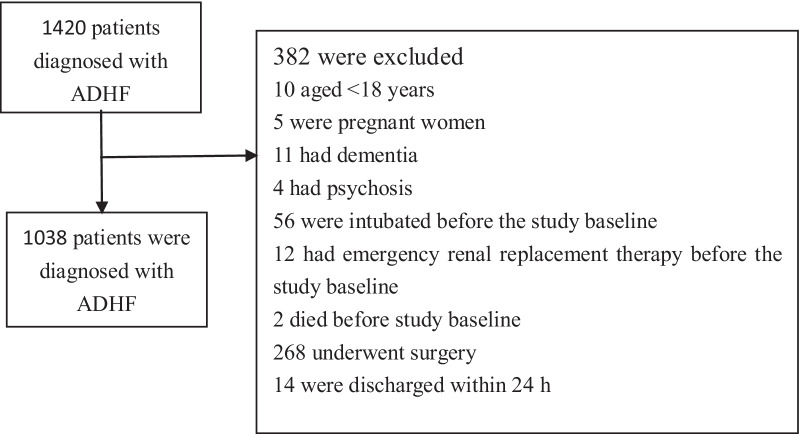
Flow chart demonstrating the patient screening process of 1420 patient samples

382 of the 1420 consecutive ADHF patients hospitalized from January 1, 2017, to February 27, 2021 were excluded from this study. Finally, 1038 patients eligible for inclusion were recruited for the study (Fig. 1).

In total, 1038 ADHF patients were recruited for this research. The average age was 78.0 years and 496 (47.8%) of the patients were men. The time from admission to albumin administration was 1.0 (1.0–2.0) days. The average time duration of albumin infusion was 3.0 (2.0–3.0) days. The average dose of albumin infusion was 30.0 (20.0–40.0) g. 357 individuals (34.4 percent) experienced a primary end-point incident during a median follow-up of 9.8 days (79 individuals died before receiving intubation or undergoing emergency renal replacement therapy, 118 were intubated and 160 had emergency renal replacement therapy).

Of the 1038 patients, 337 (32.5%) were administered albumin (median dose of treatment, 29.0 g) and 701 (67.5%) were not. Table 1 shows the distribution of patients' baseline data with albumin treatment in both the unmatched and propensity-score-matched analytic samples. Albumin exposures varied by age group, body mass index, past diagnoses, clinical signs and symptoms, laboratory testing, and administration of other drugs in the unmatched cohort. Serum albumin levels at baseline were lower in patients who received albumin treatment in contrast with those who did not receive albumin treatment (median, 37.3 g/L versus 31.7 g/L, respectively).

**Table 1:** Pre- and post-propensity-score matching features of patients receiving or not receiving albumin

Variables	Unmatched patients			Propensity-score-matched patients		
	No albumin	Albumin	Standardized difference	No albumin	Albumin	Standardized difference
N	701	337		337	337	
Sex, Male (%)	342 (48.8)	154 (45.7)	0.062	151 (44.8)	154 (45.7)	0.018
Age (%)			0.215			0.029
< 60	94 (13.4)	29 (8.6)		29 (8.6)	29 (8.6)	
60–69	104 (14.8)	41 (12.2)		39 (11.6)	41 (12.2)	
70–79	206 (29.4)	93 (27.6)		97 (28.8)	93 (27.6)	
≥ 80	297 (42.4)	174 (51.6)		172 (51.0)	174 (51.6)	
BMI (%)			0.265			0.144
< 18.5	26 (3.7)	26 (7.7)		17 (5.0)	26 (7.7)	
18.5–24.9	400 (57.1)	214 (63.5)		206 (61.1)	214 (63.5)	
25–29.9	201 (28.7)	74 (22)		89 (26.4)	74 (22.0)	
≥ 30	74 (10.6)	23 (6.8)		25 (7.4)	23 (6.8)	
Diabetes (%)	296 (42.2)	143 (42.4)	0.04	165 (48.9)	143 (42.4)	0.066
Hypertension (%)	477 (68)	241 (71.5)	0.03	236 (70.0)	241 (71.5)	0.122
Coronary artery disease (%)	455 (64.9)	250 (74.2)	0.134	241 (71.5)	250 (74.2)	0.013
Previous heart failure (%)	247 (35.2)	133 (39.5)	0.031	134 (39.7)	133 (39.5)	0.049
Atrial fibrillation (%)	284 (40.5)	138 (40.9)	0.045	129 (37.7)	138 (40.9)	0.018
Previous renal dysfunction (%)	118 (16.8)	90 (26.7)	0.006	74 (21.9)	90 (26.7)	0.079
Cerebral infarction (%)	134 (19.1)	92 (27.3)	0.06	71 (21.1)	92 (27.3)	0.028
Cancer (%)	65 (9.3)	50 (14.8)	0.023	40 (11.8)	50 (14.8)	0.058
Cirrhosis (%)	6 (0.9)	6 (1.8)	0.004	1 (0.3)	6 (1.8)	0.026
NYHA classification (%)			0.021			0.094
II	186 (26.5)	82 (24.3)		75 (22.2)	82 (24.3)	
III	329 (46.9)	165 (49)		158 (46.9)	165 (49.0)	
IV	186 (26.5)	90 (26.7)		104 (30.9)	90 (26.7)	
Paroxysmal nocturnal dyspnea (%)	117 (16.7)	53 (15.7)	0.074	58 (17.2)	53 (15.7)	0.089
Orthopnoea (%)	138 (19.7)	58 (17.2)	0.03	79 (23.4)	58 (17.2)	0.053
Heart rate (beats/min)	82 (71, 100)	83 (73, 100)	0.025	82 (70, 99)	83 (73, 100)	0.025
Systolic blood pressure (mmHg)	127 (112, 146)	128 (112, 148)	0.036	129 (113, 148)	128 (112, 148)	0.035
Diastolic blood pressure (mmHg)	71 (62, 83)	69 (59, 79)	0.183	70 (60, 82)	69 (59, 79)	0.103
Rales (> 1/2 lung fields) (%)	255 (36.4)	135 (40.1)	0.04	152 (45.1)	135 (40.0)	0.102
Jugular venous distension (%)	119 (17)	67 (19.9)	0.074	76 (22.6)	67 (19.9)	0.065
Peripheral edema (%)	434 (61.9)	239 (70.9)	0.069	229 (68.0)	239 (70.9)	0.064
LVEF (%)	54 (43, 60)	55 (45, 60)	0.064	55 (45, 60)	55 (45, 60)	0.028
B-type natriuretic peptide (pg/ml)	753 (319, 1606)	817 (384, 2148)	0.17	913 (363, 1775)	817 (384, 2148)	0.096
Troponin I(ng/ml)	0.06 (0.04, 0.1)	0.06 (0.04, 0.1)	0.011	0.06 (0.05, 0.1)	0.06 (0.04, 0.1)	0.07
Hemoglobin (g/L)	120.9 (24)	106.2 (23.6)	0.614	113.48 (23.67)	106.24 (23.6)	0.306
C-reactive protein (mg/L)	8 (3.65, 18.74)	13.08 (4.29, 40.29)	0.298	9.61 (4.07, 25.01)	13.08 (4.29, 40.29)	0.149
Alanine aminotransferase (IU/L)	17.8 (11.9, 31.6)	14.7 (9.8, 24.1)	0.147	16.5 (11.3, 29.8)	14.7 (9.8, 24.1)	0.149
Total bilirubin (μmol/L)	14.5 (10.2, 21.5)	12.7 (8.7, 18.9)	0.101	13.2 (9.4, 18.9)	12.7 (8.7, 18.9)	0.011
Blood urea nitrogen (mmol/L)	8.1 (5.8, 11.4)	8.7 (6.2, 13.4)	0.166	8.6 (6.3, 12.6)	8.7 (6.2, 13.4)	0.016
Creatinine (μmol/L)	93 (73.3, 122.1)	98.4 (71.5, 144)	0.263	99.1 (75.5, 131)	98.4 (71.5, 144)	0.131
Serum albumin (g/L)	37.3 (35.3, 39.5)	31.7 (29.2, 33.3)	1.304	35.2 (34, 36.2)	31.7 (29.2, 33.3)	0.825
Sodium (mmol/L)	138.9 (136.2, 141.4)	138.4 (135.6, 141.6)	0.027	139.1 (136.2, 141.8)	138.4 (135.6, 141.6)	0.049
Potassium (mmol/L)	4.19 (3.87, 4.6)	4.1 (3.74, 4.59)	0.123	4.25 (3.89, 4.67)	4.1 (3.74, 4.59)	0.187
Uric acid (μmol/L)	408 (312.9, 527.2)	376 (275, 503)	0.214	401.2 (312, 521.8)	376 (275, 503)	0.159
Glucose (mmol/L)	7.35 (5.85, 9.75)	7.06 (5.67, 9.8)	0.08	7.29 (5.85, 10.08)	7.06 (5.67, 9.8)	0.114
Aldosterone antagonists (%)	542 (77.3)	223 (66.2)	0.057	256 (76.0)	223 (66.2)	0.217
Loop diuretic (%)	655 (93.4)	307 (91.1)	0.153	314 (93.2)	307 (91.1)	0.077
ACE-Is/ARBs (%)	297 (42.4)	104 (30.9)	0.065	136 (40.4)	104 (30.9)	0.199
Beta-blockers (%)	496 (70.8)	225 (66.8)	0.037	233 (69.1)	225 (66.8)	0.051
Anticoagulants (%)	227 (32.4)	82 (24.3)	0.071	103 (30.6)	82 (24.3)	0.14
Aspirin (%)	301 (42.9)	137 (40.7)	0.069	134 (39.8)	137 (40.7)	0.018
Vasopressor (%)	68 (9.7)	28 (8.3)	0.08	40 (11.9)	28 (8.3)	0.118
rh-BNP (%)	34 (4.9)	16 (4.7)	0.005	11 (3.3)	16 (4.7)	0.076
NPPV (%)	123 (17.5)	95 ( 28.2)	0.255	72 (21.4)	95 (28.2)	0.159

Propensity-Score Matching covariates were sex, age, coronary artery disease, heart rate, systolic blood pressure, creatinine and serum albumin

Data are presented as frequencies (percentages) or mean (SD) or median (IQR)

*BMI* Body mass index (weight in kilograms divided by the square of height in meters), *NYHA* New York Heart Association, *LVEF* Left ventricular ejection fraction, *ACE-Is/ARBs* Angiotensin-converting enzyme inhibitors/angiotensin receptor blockers. *rh-BNP* Recombinant human brain natriuretic peptide, *NPPV* non-invasive positive pressure ventilation

### Statistical analysis

**Fig. 2:**
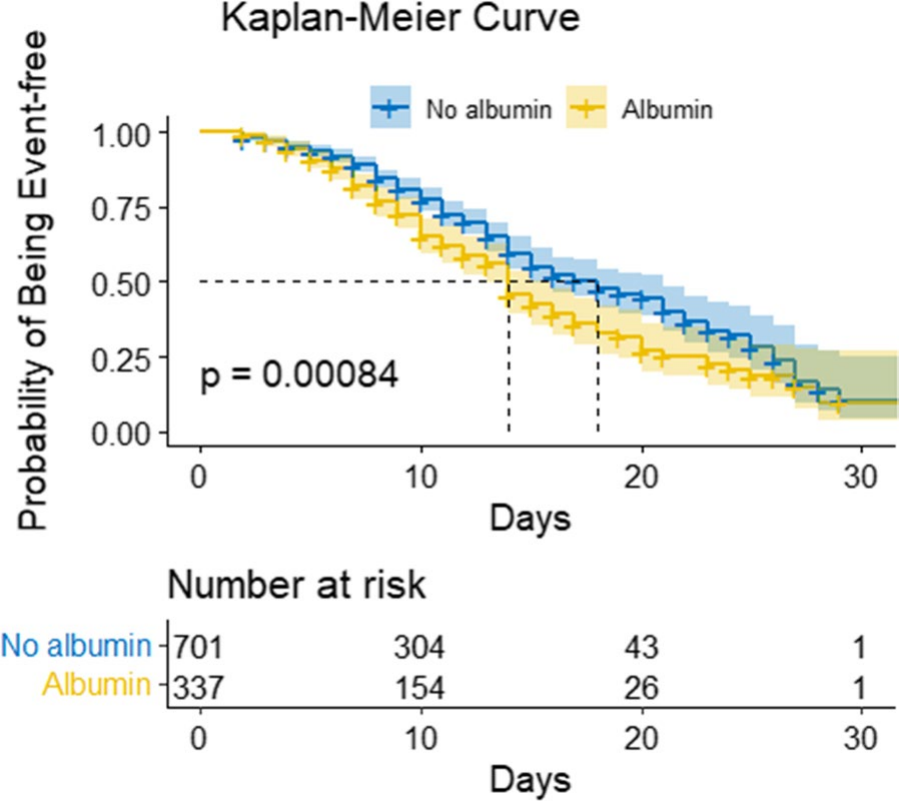
Freedom from composite end-point. Pointwise 95% confidence intervals are represented by shaded regions

The primary end-point occurred in 357 of the 1038 (34.4%) patients enrolled in the analysis. Patients who received albumin had a higher likelihood of experiencing a primary end-point incident compared to those who did not receive it, according to the crude unadjusted analysis (hazard ratio, 1.43; 95% CI 1.16–1.76) (Fig. 2). Additional file 1: Tables S5–S6 present the associations between each confounder and outcomes.

Additional file 1: Fig. S1 depicts the distribution of predicted propensity scores across patients who received and did not receive albumin administration. The C-statistic of the propensity-score model was 0.845. 337 patients were exposed but 337 were not exposed in the matched analytic cohort. In the propensity-score-matched Cox proportional hazard regression analysis, no considerable relationship was identified between albumin administration and composite primary end-point (hazard ratio, 1.11; 95% CI 0.64–1.57) (Table 2).

**Table 2:** Associations between the use of albumin and composite end-point in the propensity-score, multivariable, and crude analyses

Analysis	Composite end point
No. of events/no. of patients at risk (%)	
Albumin	149 /337(44.2)
No albumin	208/701 (29.7)
Crude analysis—hazard ratio (95% CI)	1.43(1.16–1.76)
Multivariable analysis—hazard ratio (95% CI) *	1.00(0.75–1.32)
Propensity-score analyses—hazard ratio (95% CI)	
With matching**	1.11 (0.64–1.57)
With inverse probability weighting***	1.05 (0.75–1.47)

^*^Shown is the hazard ratio from the multivariate Cox proportional-hazards model adjusted for past diagnoses, sex, clinical signs and symptoms, laboratory tests, and medications. All 1038 participants were included in the analysis

^**^ Shown is the hazard ratio from a multivariate Cox proportional-hazards model with identical strata and covariates matching on the basis of the propensity score. 674 patients were involved in the analysis (337 were treated with albumin and 337 were not)

^***^Shown is the primary analysis with a hazard ratio from the IPW multivariate Cox proportional hazards model with identical strata and covariates on the basis of the propensity score. All patients were involved in the analysis

In the multivariable IPW analysis, according to the propensity score, no considerable relationship was identified between the use of albumin and the composite primary end-point (hazard ratio, 1.05; 95% CI 0.75–1.47) (Table 2). Furthermore, a nonparametric bootstrap was created to construct CIs for the IPW Kaplan–Meier curves (Additional file 1: Fig. S2).

### Sensitivity analysis

The risk of intubation, emergency renal replacement, or mortality was similar amongst patients who were administered albumin versus those without albumin administration with different matching strategies (hazard ratio, 1.14; 95% CI 0.97–1.58 and 1.07; 95% CI 0.64–1.30) (Additional file 1: Table S7). In the sub-group analysis, the administration of albumin did not show a relationship with either a greatly reduced or aggregated risk of the composite end-point (Additional file 1: Table S8).

## Discussion

In this research including a large number of consecutive hospitalized AHF patients, the risk of intubation, emergency renal replacement, or mortality was neither substantially higher nor lower amongst patients who were administered albumin versus those without albumin administration (hazard ratio, 1.05; 95% CI 0.75–1.47). Considering the observational methodology and larger confidence intervals used in this study, the benefits or risks of albumin administration should not be disregarded. Our findings, however, do not justify the usage of albumin at this time.

Hypoalbuminaemia commonly occurs in hospitalized patients and is particularly prevalent among AHF patients. Amongst these patients, hypoalbuminaemia is closely linked to mortality [1, 2, 18]. Intravenous albumin can be applied to avert or manage hypotension or to assist with fluid drainage. However, this is a contentious technique [19, 20]. Although intravenous albumin is widely thought to be safe, it is also quite costly. The impacts of intravenous administration of 20% albumin on volume needs, fluid balance, biochemical, and physiological responses are yet to be comprehensively evaluated in randomized control trials. Furthermore, potential risks are used for preventing or managing intradialytic hypotension. Several research reports examined the potential advantages and drawbacks of albumin treatment through intravenous injection to reduce intradialytic hypotension and/or improve ultrafiltration [3, 5]. Earlier studies on the efficacy of albumin solutions in resuscitation predominantly focused on critically ill and patients with sepsis and hypoalbuminaemia [10, 11, 13–15]. No previous studies have focussed on albumin use for patients with AHF.

We strived to minimize any confounding factors in a variety of approaches with the analytic tools utilized in this study of our observational sample. There was no considerable connection between albumin usage and the intubation risk, emergency renal replacement, or mortality in the primary analysis, with an IPW multivariable regression model based on the propensity score. Analyses were conducted using several propensity-score approaches. Encouragingly, the outcomes of these analyses are consistent. In this research, possible confounders, such as sex, diabetes, coronary artery disease, previous renal dysfunction, clinical signs and symptoms, laboratory tests, and medication use, were adjusted. To avoid immortal time bias, the baseline for the study was set as 24 h before the albumin administration.

### Limitations

We acknowledge various limitations in this study. First, this was an observational study, and a cohort of patients with AHF was evaluated. The patients with chronic stable heart failure were excluded; therefore, this population could not be extrapolated. Second, some information, such as a myocardial infarction history and the etiology of heart failure, were not considered for the analysis, and nonlinearity and interaction of variables were not analyzed. Lastly, these results may be limited in their generalisability due to the single-center design.

## Conclusion

In this research including a number of consecutive hospitalized AHF patients, albumin usage was found to have no association with a greatly increased or reduced risk of intubation, emergency renal replacement, or mortality (hazard ratio, 1.05; 95% CI 0.75–1.47). The findings from this research should not exclude the benefits or risks of albumin administration, considering the observational methodology and 95% CI. Nonetheless, our findings do not advocate the current use of albumin, and further randomized clinical trials should be conducted to evaluate its efficacy.

## Supplementary Information

**Additional file 1: Table S1.** Definition of variables. **Table S2.** Baseline characteristics of 1038 patients: pre-imputation and post-imputation. **Table S3.** Comparison of the characteristics between the Albumin and No albumin groups. **Table S4.** Comparison of the characteristics between the Albumin and No albumin groups after Propensity-Score Matching. **Table S5.** Univariate cox regression model. **Table S6.** Multivariable cox regression model. **Table S7.** Propensity-score matching features of patients receiving or not receiving albumin with different matching strategies. **Table S8.** Sub-group analysis. **Fig. S1.** Distribution of the estimated propensity score for receiving albumin, among patients who did and did not actually receive the treatment. **Fig. S2.** Freedom from Composite End Point with inverse probability weighting according to the propensity score. The shaded areas represent pointwise 95% confidence intervals.

## Data Availability

The datasets used and/or analyzed during the current study are available from the corresponding author on reasonable request.
