# Supplementary material for: Albumin infusion in hospitalized patients with acute heart failure: a retrospective cohort study
Source: BMC Cardiovasc Disord. 2022 Aug 6;22:358. doi: 10.1186/s12872-022-02797-1 (PMC9356412; doi:10.1186/s12872-022-02797-1)
Supplement: Supplementary file 1 — Additional file 1: Table S1. Definition of variables. Table S2. Baseline characteristics of 1038 patients: pre-imputation and post-imputation. Table S3. Comparison of the characteristics between the Albumin and No albumin groups. Table S4. Comparison of the characteristics between the Albumin and No albumin groups after Propensity-Score Matching. Table S5. Univariate cox regression model. Table S6. Multivariable cox regression model. Table S7. Propensity-score matching features of patients receiving or not receiving albumin with different matching strategies. Table S8. Sub-group analysis. Fig. S1. Distribution of the estimated propensity score for receiving albumin, among patients who did and did not actually receive the treatment. Fig. S2. Freedom from Composite End Point with inverse probability weighting according to the propensity score. The shaded areas represent pointwise 95% confidence intervals. [file 12872_2022_2797_MOESM1_ESM.doc]

Supplemental Material

Table S1. Definition of variables.

| Variables | Method of assessment and grading | Reference |
| --- | --- | --- |
| Demographic information |  |  |
| Sex | Female  Male | Female vs male |
| Age | Assessed at admission | In units of years |
| Height | Assessed at admission | In units of m |
| Weight | Assessed at admission | In units of Kg |
| Medical History |  |  |
| Diabetes | Diabetes mellitus, Type 1 or 2 diabetes, taking hypoglycemic medications | Absence vs presence |
| Hypertension | Hypertension, primary or secondary Hypertension, hypertension grade 1, 2 or 3 | Absence vs presence |
| Coronary artery disease | Coronary artery disease, angina, myocardial infarction, ischemic heart disease, coronary artery stent implantation | Absence vs presence |
| Previous heart failure | Heart failure, systolic heart failure, diastolic heart failure, cardiac dysfunction, heart failure unspecified | Absence vs presence |
| Atrial fibrillation | Atrial fibrillation, paroxysmal or persistent atrial fibrillation | Absence vs presence |
| Previous renal dysfunction | Renal dysfunction, renal failure, renal failure unspecified | Absence vs presence |
| Cerebral infarction | Cerebral infarction, cerebral stroke | Absence vs presence |
| Cancer | Cancer, tumors of the organs or blood systems | Absence vs presence |
| Cirrhosis | Liver cirrhosis, compensatory or decompensated cirrhosis | Absence vs presence |
| Clinical information |  |  |
| NYHA classification | Assessed according to the NYHA Functional Classification  I: Cardiac disease, but no symptoms and no limitation in ordinary physical activity, e.g. no  shortness of breath when walking, climbing stairs etc.  II: Mild symptoms (mild shortness of breath and/or angina) and slight limitation during  ordinary activity.  III: Marked limitation in activity due to symptoms, even during less-than-ordinary activity,  e.g. walking short distances (20–100 m); comfortable only at rest.  IV: Severe limitations. Experiences symptoms even while at rest. | II vs III vs IV |
| Paroxysmal nocturnal dyspnea | Assessed at baseline | Absence vs presence |
| Orthopnoea | Assessed at baseline | Absence vs presence |
| Heart rate | Assessed at baseline | In units of beats / minute |
| Systolic blood pressure | Assessed at baseline | In units of mmHg |
| Diastolic blood pressure | Assessed at baseline | In units of mmHg |
| Rales (>1/2 lung fields) | Assessed at baseline | Absence vs presence |
| Jugular venous distension | Assessed at baseline | Absence vs presence |
| Peripheral edema | Assessed at baseline | Absence vs presence |
| Imaging results |  |  |
| LVEF | The first value at baseline by two-dimensional transthoracic echocardiography | In units of % |
| Laboratory findings |  |  |
| B-type natriuretic peptide | The first value at baseline | In units of pg/ml |
| Troponin I | The first value at baseline | In units of ng/ml |
| Hemoglobin | The first value at baseline | In units of g/L |
| C-reactive protein | The first value at baseline | In units of mg/L |
| Alanine aminotransferase | The first value at baseline | In units of IU/L |
| Total bilirubin | The first value at baseline | In units of (μmol/L) |
| Blood urea nitrogen | The first value at baseline | In units of mmol/L |
| Creatinine | The first value at baseline | In units of μmol/L |
| Serum albumin | The first value at baseline | In units of g/L |
| Serum sodium | The first value at baseline | In units of mmol/L |
| Serum potassium | The first value at baseline | In units of mmol/L |
| Uric acid | The first value at baseline | In units of μmol/L |
| Glucose | The first value at baseline | In units of mmol/L |
| Treatment |  |  |
| Aldosterone antagonists | Assessed at baseline | Used or not used |
| Loop diuretic | Assessed at baseline | Used or not used |
| ACE-Is/ARBs | Assessed at baseline | Used or not used |
| Beta-blockers | Assessed at baseline | Used or not used |
| Anticoagulants | Assessed at baseline | Used or not used |
| Aspirin | Assessed at baseline | Used or not used |
| Vasopressor | Assessed at baseline | Used or not used |
| rh-BNP | Assessed at baseline | Used or not used |
| NPPV | Assessed at baseline | Used or not used |
| Albumin infusion | During the period of observation | The time from admission to albumin infusion and the time duration and doses |

Variables were chosen based on a previous literature review and clinical evidence of risk factors for mortality, hospitalization for heart failure.

History of Medical History recorded using diagnosis codes (International Classification of Diseases, Ninth Revision, Clinical Modification.

Abbreviations:

NYHA, New York Heart Association

LVEF, left ventricular ejection fraction

ACE-Is/ARBs, angiotensin-converting enzyme inhibitors / Angiotensin Receptor Blockers

rh-BNP, recombinant human brain natriuretic peptide

NPPV, non-invasive positive pressure ventilation

## Table S2. Baseline characteristics of 1038 patients: pre-imputation and post-imputation.

| Variables | Pre-imputation | Post-imputation |
| --- | --- | --- |
| Sex, Male (%) | 496 (47.8) | 496 (47.8) |
| Age, years | 78.0 (69.0, 84.0) | 78.0 (69.0, 84.0) |
| BMI (kg/m2) | 23.6 (21.8, 26.7) | 23.6 (21.8, 26.7) |
| Missing (%) | 41(3.9) |  |
| Diabetes (%) | 439 (42.3) | 439 (42.3) |
| Hypertension (%) | 718 (69.2) | 718 (69.2) |
| Coronary artery disease (%) | 705 (67.9) | 705 (67.9) |
| Previous heart failure (%) | 380 (36.6) | 380 (36.6) |
| Atrial fibrillation (%) | 422 (40.7) | 422 (40.7) |
| Previous renal dysfunction (%) | 208 (20.0) | 208 (20.0) |
| Cerebral infarction (%) | 226(21.8) | 226(21.8) |
| Cancer (%) | 115 (11.1) | 115 (11.1) |
| Cirrhosis (%) | 12 (1.2) | 12 (1.2) |
| NYHA classification (%) |  |  |
| II | 268 (25.8) | 268 (25.8) |
| III | 494 (47.6) | 494 (47.6) |
| IV | 276 (26.6) | 276 (26.6) |
| Paroxysmal nocturnal dyspnea (%) | 170 (16.4) | 170 (16.4) |
| Orthopnoea (%) | 196 (18.9) | 196 (18.9) |
| Heart rate (beats/min) | 82.0 (71.3, 100.0) | 82.0 (71.3, 100.0) |
| Systolic blood pressure (mmHg) | 128.0 (112.0, 147.0) | 128.0 (112, 147.0) |
| Diastolic blood pressure (mmHg) | 70.0 (61.0, 81.8) | 70.0 (61.0, 81.8) |
| Rales (>1/2 lung fields) (%) | 390 (37.6) | 390 (37.6) |
| Jugular venous distension (%) | 186 (17.8) | 186 (17.8) |
| Peripheral edema (%) | 673(64.8) | 673(64.8) |
| LVEF (%) | 55.0 (44.0, 60.0) | 55.0 (44.0, 60.0) |
| Missing (%) | 1(0.1) |  |
| B-type natriuretic peptide (pg/ml) | 782.5 (334.5, 1719.5) | 782.5 (334.5, 1719.5) |
| Missing (%) | 20(1.9) |  |
| Troponin I(ng/ml) | 0.06 (0.04, 0.1) | 0.06 (0.04, 0.1) |
| Hemoglobin (g/L) | 116.1 (24.8) | 116.1 (24.8) |
| C-reactive protein (mg/L) | 9.1 (3.9, 25.6) | 9.0 (3.9, 24.9) |
| Missing (%) | 89(8.6) |  |
| Alanine aminotransferase (IU/L) | 16.6 (11.3, 28.4) | 16.6 (11.3, 28.4) |
| Missing (%) | 6(0.6) |  |
| Total bilirubin (μmol/L) | 13.9 (9.9, 20.8) | 13.9 (9.9, 20.7) |
| Missing (%) | 7(0.7) |  |
| Blood urea nitrogen (mmol/L) | 8.2 (5.9, 11.8) | 8.2 (5.9, 11.8) |
| Creatinine (μmol/L) | 93.9 (72.9, 128.4) | 93.9 (72.9, 128.4) |
| Serum albumin (g/L) | 35.9 (32.8, 38.8) | 35.9 (32.8, 38.8) |
| Missing (%) | 5(0.5) |  |
| Sodium (mmol/L) | 138.7 (136.0, 141.5) | 138.7 (136.0, 141.5) |
| Potassium (mmol/L) | 4.2 (3.8, 4.6) | 4.2 (3.8, 4.6) |
| Uric acid (μmol/L) | 400.7 (303.7, 521.0) | 400.7 (303.7, 521.0) |
| Missing (%) | 5(0.5) |  |
| Glucose (mmol/L) | 7.3 (5.7, 9.8) | 7.3 (5.7, 9.8) |
| Missing (%) | 5(0.5) |  |
| Aldosterone antagonists (%) | 765(73.7) | 765(73.7) |
| Loop diuretic (%) | 962 (92.7) | 962 (92.7) |
| ACE-Is/ARBs (%) | 401(38.6) | 401(38.6) |
| Beta-blockers (%) | 721 (69.5) | 721 (69.5) |
| Anticoagulants (%) | 309 (29.8) | 309 (29.8) |
| Aspirin (%) | 309 (41.7) | 309 (41.7) |
| Vasopressor (%) | 60 (9.2) | 60 (9.2) |
| rh-BNP (%) | 50 ( 4.8) | 50 ( 4.8) |
| NPPV (%) | 218 (21.0) | 218 (21.0) |

History of diabetes or hypertension recorded using diagnosis codes (International Classification of Diseases, Ninth Revision, Clinical Modification.

Data are presented as frequencies (percentages) or mean (SD) or median (IQR)

Abbreviations:

BMI, the body-mass index, the weight in kilograms divided by the square of the height in meters.

NYHA, New York Heart Association

LVEF, left ventricular ejection fraction

ACE-Is/ARBs, angiotensin-converting enzyme inhibitors / Angiotensin Receptor Blockers

rh-BNP, recombinant human brain natriuretic peptide

NPPV, non-invasive positive pressure ventilationSD, standard deviation

IQR, interquartile range

Table S3 Comparison of the characteristics between the Albumin and No albumin groups

| Variables | No albumin | Albumin | P-value |
| --- | --- | --- | --- |
| N (%) | 701 (67.5) | 337 (32.5) |  |
| Sex, Male (%) | 342 (48.8) | 154 (45.7) | 0.39 |
| Age (%) |  |  | 0.02 |
| <60 | 94 (13.4) | 29 (8.6) |  |
| 60–69 | 104 (14.8) | 41 (12.2) |  |
| 70–79 | 206 (29.4) | 93 (27.6) |  |
| ≥80 | 297 (42.4) | 174 (51.6) |  |
| BMI (%) |  |  | < 0.01 |
| <18.5 | 26 (3.7) | 26 (7.7) |  |
| 18.5–24.9 | 400 (57.1) | 214 (63.5) |  |
| 25–29.9 | 201 (28.7) | 74 (22) |  |
| ≥30 | 74 (10.6) | 23 (6.8) |  |
| Diabetes (%) | 296 (42.2) | 143 (42.4) | 1 |
| Hypertension (%) | 477 (68) | 241 (71.5) | 0.29 |
| Coronary artery disease (%) | 455 (64.9) | 250 (74.2) | < 0.01 |
| Previous heart failure (%) | 247 (35.2) | 133 (39.5) | 0.21 |
| Atrial fibrillation (%) | 284 (40.5) | 138 (40.9) | 0.95 |
| Previous renal dysfunction (%) | 118 (16.8) | 90 (26.7) | < 0.01 |
| Cerebral infarction (%) | 134 (19.1) | 92 (27.3) | < 0.01 |
| Cancer (%) | 65 (9.3) | 50 (14.8) | 0.01 |
| Cirrhosis (%) | 6 (0.9) | 6 (1.8) | 0.22 |
| NYHA classification (%) |  |  | 0.73 |
| II | 186 (26.5) | 82 (24.3) |  |
| III | 329 (46.9) | 165 (49) |  |
| IV | 186 (26.5) | 90 (26.7) |  |
| Paroxysmal nocturnal dyspnea (%) | 117 (16.7) | 53 (15.7) | 0.76 |
| Orthopnoea (%) | 138 (19.7) | 58 (17.2) | 0.38 |
| Heart rate (beats/min) | 82 (71, 100) | 83 (73, 100) | 0.91 |
| Systolic blood pressure (mmHg) | 127 (112, 146) | 128 (112, 148) | 0.7 |
| Diastolic blood pressure (mmHg) | 71 (62, 83) | 69 (59, 79) | < 0.01 |
| Rales (>1/2 lung fields) (%) | 255 (36.4) | 135 (40.1) | 0.28 |
| Jugular venous distension (%) | 119 (17) | 67 (19.9) | 0.29 |
| Peripheral edema (%) | 434 (61.9) | 239 (70.9) | < 0.01 |
| LVEF (%) | 54 (43, 60) | 55 (45, 60) | 0.36 |
| B-type natriuretic peptide (pg/ml) | 753 (319, 1606) | 817 (384, 2148) | 0.02 |
| Troponin I(ng/ml) | 0.06 (0.04, 0.1) | 0.06 (0.04, 0.1) | 0.72 |
| Hemoglobin (g/L) | 120.9 (24) | 106.2 (23.6) | < 0.01 |
| C-reactive protein (mg/L) | 8 (3.65, 18.74) | 13.08 (4.29, 40.29) | < 0.01 |
| Alanine aminotransferase (IU/L) | 17.8 (11.9, 31.6) | 14.7 (9.8, 24.1) | < 0.01 |
| Total bilirubin (μmol/L) | 14.5 (10.2, 21.5) | 12.7 (8.7, 18.9) | < 0.01 |
| Blood urea nitrogen (mmol/L) | 8.1 (5.8, 11.4) | 8.7 (6.2, 13.4) | 0.01 |
| Creatinine (μmol/L) | 93 (73.3, 122.1) | 98.4 (71.5, 144) | 0.04 |
| Serum albumin (g/L) | 37.3 (35.3, 39.5) | 31.7 (29.2, 33.3) | < 0.01 |
| Sodium (mmol/L) | 138.9 (136.2, 141.4) | 138.4 (135.6, 141.6) | 0.34 |
| Potassium (mmol/L) | 4.19 (3.87, 4.6) | 4.1 (3.74, 4.59) | 0.07 |
| Uric acid (μmol/L) | 408 (312.9, 527.2) | 376 (275, 503) | < 0.01 |
| Glucose (mmol/L) | 7.35 (5.85, 9.75) | 7.06 (5.67, 9.8) | 0.41 |
| Aldosterone antagonists (%) | 542 (77.3) | 223 (66.2) | < 0.01 |
| Loop diuretic (%) | 655 (93.4) | 307 (91.1) | 0.22 |
| ACE-Is/ARBs (%) | 297 (42.4) | 104 (30.9) | < 0.01 |
| Beta-blockers (%) | 496 (70.8) | 225 (66.8) | 0.22 |
| Anticoagulants (%) | 227 (32.4) | 82 (24.3) | < 0.01 |
| Aspirin (%) | 301 (42.9) | 137 (40.7) | 0.53 |
| Vasopressor (%) | 68 (9.7) | 28 (8.3) | 0.54 |
| rh-BNP (%) | 34 (4.9) | 16 (4.7) | 1.00 |
| NPPV (%) | 123 (17.5) | 95 ( 28.2) | < 0.01 |

P-value: Means for continuous variables were compared using t tests when the data were normally distributed; otherwise, the Mann-Whitney U test was used. Proportions for categorical variables were compared using the χ2 test, although the Fisher exact probability test was used when the data were limited.

Abbreviations:

BMI, the body-mass index, the weight in kilograms divided by the square of the height in meters.

NYHA, New York Heart Association

LVEF, left ventricular ejection fraction

ACE-Is/ARBs, angiotensin-converting enzyme inhibitors / Angiotensin Receptor Blockers

rh-BNP, recombinant human brain natriuretic peptide

NPPV, non-invasive positive pressure ventilation

Table S4 Comparison of the characteristics between the Albumin and No albumin groups after Propensity-Score Matching

| Variables | No albumin | Albumin | P-value |
| --- | --- | --- | --- |
| N (%) | 337 | 337 |  |
| Sex, Male (%) | 151 (44.8) | 154 (45.7) | 0.877 |
| Age (%) |  |  | 0.99 |
| <60 yr | 29 (8.6) | 29 (8.6) |  |
| 60–69 yr | 39 (11.6) | 41 (12.2) |  |
| 70–79 yr | 97 (28.8) | 93 (27.6) |  |
| ≥80 yr | 172 (51.0) | 174 (51.6) |  |
| BMI (%) |  |  | 0.32 |
| <18.5 | 17 (5.0) | 26 (7.7) |  |
| 18.5–24.9 | 206 (61.1) | 214 (63.5) |  |
| 25–29.9 | 89 (26.4) | 74 (22.0) |  |
| ≥30 | 25 (7.4) | 23 (6.8) |  |
| Diabetes (%) | 165 (48.9) | 143 (42.4) | 0.104 |
| Hypertension (%) | 236 (70.0) | 241 (71.5) | 0.735 |
| Coronary artery disease (%) | 241 (71.5) | 250 (74.2) | 0.488 |
| Previous heart failure (%) | 134 (39.7) | 133 (39.5) | 1 |
| Atrial fibrillation (%) | 129 (37.7) | 138 (40.9) | 0.529 |
| Previous renal dysfunction (%) | 74 (21.9) | 90 (26.7) | 0.178 |
| Cerebral infarction (%) | 71 (21.1) | 92 (27.3) | 0.072 |
| Cancer (%) | 40 (11.8) | 50 (14.8) | 0.308 |
| Cirrhosis (%) | 1 (0.3) | 6 (1.8) | 0.123 |
| NYHA classification (%) |  |  | 0.479 |
| II | 75 (22.2) | 82 (24.3) |  |
| III | 158 (46.9) | 165 (49.0) |  |
| IV | 104 (30.9) | 90 (26.7) |  |
| Paroxysmal nocturnal dyspnea (%) | 58 (17.2) | 53 (15.7) | 0.678 |
| Orthopnoea (%) | 79 (23.4) | 58 (17.2) | 0.056 |
| Heart rate (beats/min) | 82 (70, 99) | 83 (73, 100) | 0.322 |
| Systolic blood pressure (mmHg) | 129 (113, 148) | 128 (112, 148) | 0.605 |
| Diastolic blood pressure (mmHg) | 70 (60, 82) | 69 (59, 79) | 0.189 |
| Rales (>1/2 lung fields) (%) | 152 (45.1) | 135 (40.0) | 0.213 |
| Jugular venous distension (%) | 76 (22.6) | 67 (19.9) | 0.451 |
| Peripheral edema (%) | 229 (68.0) | 239 (70.9) | 0.452 |
| LVEF (%) | 55 (45, 60) | 55 (45, 60) | 0.93 |
| B-type natriuretic peptide (pg/ml) | 913 (363, 1775) | 817 (384, 2148) | 0.525 |
| Troponin I(ng/ml) | 0.06 (0.05, 0.1) | 0.06 (0.04, 0.1) | 0.18 |
| Hemoglobin (g/L) | 113.48 (23.67) | 106.24 (23.6) | < 0.001 |
| C-reactive protein (mg/L) | 9.61 (4.07, 25.01) | 13.08 (4.29, 40.29) | 0.046 |
| Alanine aminotransferase (IU/L) | 16.5 (11.3, 29.8) | 14.7 (9.8, 24.1) | 0.015 |
| Total bilirubin (μmol/L) | 13.2 (9.4, 18.9) | 12.7 (8.7, 18.9) | 0.518 |
| Blood urea nitrogen (mmol/L) | 8.6 (6.3, 12.6) | 8.7 (6.2, 13.4) | 0.809 |
| Creatinine (μmol/L) | 99.1 (75.5, 131) | 98.4 (71.5, 144) | 0.759 |
| Serum albumin (g/L) | 35.2 (34, 36.2) | 31.7 (29.2, 33.3) | < 0.001 |
| Sodium (mmol/L) | 139.1 (136.2, 141.8) | 138.4 (135.6, 141.6) | 0.295 |
| Potassium (mmol/L) | 4.25 (3.89, 4.67) | 4.1 (3.74, 4.59) | 0.012 |
| Uric acid (μmol/L) | 401.2 (312, 521.8) | 376 (275, 503) | 0.023 |
| Glucose (mmol/L) | 7.29 (5.85, 10.08) | 7.06 (5.67, 9.8) | 0.364 |
| Aldosterone antagonists (%) | 256 (76.0) | 223 (66.2) | 0.007 |
| Loop diuretic (%) | 314 (93.2) | 307 (91.1) | 0.391 |
| ACE-Is/ARBs (%) | 136 (40.4) | 104 (30.9) | 0.013 |
| Beta-blockers (%) | 233 (69.1) | 225 (66.8) | 0.563 |
| Anticoagulants (%) | 103 (30.6) | 82 (24.3) | 0.084 |
| Aspirin (%) | 134 (39.8) | 137 (40.7) | 0.875 |
| Vasopressor (%) | 40 (11.9) | 28 (8.3) | 0.159 |
| rh-BNP (%) | 11 (3.3) | 16 (4.7) | 0.432 |
| NPPV (%) | 72 (21.4) | 95 (28.2) | 0.05 |

Propensity-Score Matching covariates were sex, age, coronary artery disease, heart rate, systolic blood pressure, creatinine and serum albumin.

P-value: Means for continuous variables were compared using t tests when the data were normally distributed; otherwise, the Mann-Whitney U test was used. Proportions for categorical variables were compared using the χ2 test, although the Fisher exact probability test was used when the data were limited.

Abbreviations:

BMI, the body-mass index, the weight in kilograms divided by the square of the height in meters.

NYHA, New York Heart Association

LVEF, left ventricular ejection fraction

ACE-Is/ARBs, angiotensin-converting enzyme inhibitors / Angiotensin Receptor Blockers

rh-BNP, recombinant human brain natriuretic peptide

NPPV, non-invasive positive pressure ventilation

Table S5 Univariate cox regression model

| Variables | HR | CI | P-value |
| --- | --- | --- | --- |
| Albumin group | 1.43 | 1.16-1.76 | 0.001 |
| Sex, Female | 0.76 | 0.62-0.94 | 0.01 |
| Age |  |  |  |
| <60 yr |  |  |  |
| 60–69 yr | 0.81 | 0.51-1.28 | 0.362 |
| 70–79 yr | 1.02 | 0.7-1.5 | 0.899 |
| ≥80 yr | 1.07 | 0.74-1.53 | 0.728 |
| BMI (kg/m2) |  |  |  |
| <18.5 |  |  |  |
| 18.5–24.9 | 1.32 | 0.75-2.31 | 0.337 |
| 25–29.9 | 1.26 | 0.7-2.26 | 0.441 |
| ≥30 | 1.07 | 0.54-2.12 | 0.84 |
| Diabetes (%) | 1.24 | 1.01-1.53 | 0.041 |
| Hypertension (%) | 1.04 | 0.83-1.3 | 0.739 |
| Coronary artery disease (%) | 1.74 | 1.35-2.23 | 0 |
| Previous heart failure (%) | 1.1 | 0.89-1.36 | 0.359 |
| Atrial fibrillation (%) | 0.86 | 0.69-1.07 | 0.177 |
| Previous renal dysfunction (%) | 1.73 | 1.38-2.15 | 0 |
| Cerebral infarction (%) | 1.13 | 0.88-1.46 | 0.323 |
| Cancer (%) | 1.33 | 0.98-1.8 | 0.071 |
| Cirrhosis (%) | 0.45 | 0.11-1.8 | 0.259 |
| NYHA classification (%) |  |  |  |
| II | 1 |  |  |
| III | 1.67 | 1.34 - 2.07 | 0 |
| IV | 1.03 | 0.86 - 1.23 | 0.762 |
| Paroxysmal nocturnal dyspnea (%) | 1.11 | 0.85-1.46 | 0.432 |
| Orthopnoea (%) | 1.6 | 1.28-2.02 | 0 |
| Heart rate (beats/min) | 1.01 | 1-1.01 | 0.001 |
| Systolic blood pressure (mmHg) | 1.01 | 1-1.01 | 0.002 |
| Diastolic blood pressure (mmHg) | 1 | 1-1.01 | 0.605 |
| Rales (>1/2 lung fields) (%) | 1.81 | 1.47-2.23 | 0 |
| Jugular venous distension (%) | 1.44 | 1.14-1.82 | 0.002 |
| Peripheral edema (%) | 1.2 | 0.95-1.51 | 0.132 |
| LVEF (%) | 0.99 | 0.98-1 | 0.112 |
| B-type natriuretic peptide (pg/ml) | 1 | 1-1.01 | 0 |
| Troponin I(ng/ml) | 1 | 0.98-1.02 | 0.802 |
| Hemoglobin (g/L) | 0.99 | 0.99-0.99 | 0 |
| C-reactive protein (mg/L) | 1 | 1-1.01 | 0.003 |
| Alanine aminotransferase (IU/L) | 1 | 1-1.01 | 0 |
| Total bilirubin | 1 | 0.99-1.01 | 0.872 |
| Blood urea nitrogen (mmol/L) | 1.05 | 1.03-1.06 | 0 |
| Creatinine (μmol/L) | 1 | 1-1.01 | 0 |
| Serum albumin (g/L) | 0.93 | 0.91-0.95 | 0 |
| Sodium (mmol/L) | 1.01 | 0.99-1.03 | 0.423 |
| Potassium (mmol/L) | 1.37 | 1.18-1.6 | 0 |
| Uric acid (μmol/L) | 1 | 1-1.01 | 0.02 |
| Glucose (mmol/L) | 1.01 | 0.98-1.03 | 0.619 |
| Aldosterone antagonists (%) | 0.61 | 0.49-0.77 | 0 |
| Loop diuretic (%) | 0.5 | 0.34-0.73 | 0 |
| ACE-Is/ARBs (%) | 0.68 | 0.54-0.86 | 0.001 |
| Beta-blockers (%) | 0.69 | 0.56-0.86 | 0.001 |
| Anticoagulants (%) | 0.79 | 0.62-1 | 0.05 |
| Aspirin (%) | 0.92 | 0.74-1.13 | 0.43 |
| Vasopressor (%) | 1.9 | 1.45-2.49 | 0 |
| rh-BNP (%) | 1.17 | 0.76-1.78 | 0.476 |
| NPPV (%) | 4.11 | 3.33-5.06 | 0.550 |

Abbreviations:

HR, hazard ratio

CI, confidence interval

BMI, the body-mass index, the weight in kilograms divided by the square of the height in meters.

NYHA, New York Heart Association

LVEF, left ventricular ejection fraction

ACE-Is/ARBs, angiotensin-converting enzyme inhibitors / Angiotensin Receptor Blockers

rh-BNP, recombinant human brain natriuretic peptide

NPPV, non-invasive positive pressure ventilation

Table S6 Multivariable cox regression model

| Variables | HR | CI | P-value |
| --- | --- | --- | --- |
| Albumin group | 1 | 0.75 - 1.32 | 0.979 |
| Sex, Female | 0.81 | 0.65 - 1.01 | 0.0597 |
| Diabetes (%) | 1.15 | 0.92 - 1.45 | 0.2216 |
| Coronary artery disease (%) | 1.45 | 1.1 - 1.9 | 0.0076 |
| Previous renal dysfunction (%) | 0.84 | 0.63 - 1.12 | 0.2322 |
| NYHA classification (%) |  |  |  |
| II | 1 |  |  |
| III | 0.99 | 0.72 - 1.35 | 0.9393 |
| IV | 0.89 | 0.71 - 1.11 | 0.3074 |
| Orthopnoea (%) | 1.36 | 0.91 - 2.03 | 0.1338 |
| Heart rate (beats/min) | 1.01 | 1 - 1.01 | 0.0001 |
| Systolic blood pressure (mmHg) | 1.01 | 1 - 1.01 | 0.0006 |
| Rales (>1/2 lung fields) (%) | 1.51 | 1.16 - 1.97 | 0.0025 |
| Jugular venous distension (%) | 0.78 | 0.58 - 1.04 | 0.0891 |
| B-type natriuretic peptide (pg/ml) | 1 | 1 - 1.01 | 0.3557 |
| Hemoglobin (g/L) | 1 | 0.99 - 1 | 0.0829 |
| C-reactive protein (mg/L) | 1 | 1 - 1.01 | 0.9741 |
| Alanine aminotransferase (IU/L) | 1 | 1 - 1.01 | 0.0294 |
| Blood urea nitrogen (mmol/L) | 1.02 | 0.99 - 1.04 | 0.1845 |
| Creatinine (μmol/L) | 1 | 1 - 1.01 | 0.0023 |
| Serum albumin (g/L) | 0.96 | 0.93 - 0.99 | 0.0124 |
| Potassium (mmol/L) | 1 | 0.83 - 1.2 | 0.9735 |
| Uric acid (μmol/L) | 1 | 1 - 1.01 | 0.1225 |
| Aldosterone antagonists (%) | 0.64 | 0.49 - 0.83 | 0.0009 |
| Loop diuretic (%) | 0.92 | 0.59 - 1.45 | 0.7278 |
| ACE-Is/ARBs (%) | 0.9 | 0.7 - 1.16 | 0.4062 |
| Beta-blockers (%) | 0.66 | 0.52 - 0.83 | 0.0006 |
| Vasopressor (%) | 1.75 | 1.28 - 2.39 | 0.0005 |

Abbreviations:

HR, hazard ratio

CI, confidence interval

BMI, the body-mass index, the weight in kilograms divided by the square of the height in meters.

NYHA, New York Heart Association

ACE-Is/ARBs, angiotensin-converting enzyme inhibitors / Angiotensin Receptor Blockers

Table S7 Propensity-score matching features of patients receiving or not receiving albumin with different matching strategies

| Variables | Matching strategy 1 | | | Matching strategy 2 | | |
| --- | --- | --- | --- | --- | --- | --- |
|  | No albumin | Albumin | Standardized difference | No albumin | Albumin | Standardized difference |
| N | 337 | 337 |  | 169 | 169 |  |
| Sex, Male (%) | 154 (45.7) | 154 (45.7) | 0 | 78 (46.2) | 80 (47.3) | 0.024 |
| Age (%) |  |  | 0.076 |  |  | 0.088 |
| <60 | 36 ( 10.7) | 29 ( 8.6) |  | 20 (11.8) | 18 ( 10.7) |  |
| 60–69 | 37 ( 11.0) | 41 ( 12.2) |  | 18 (10.7) | 15 (8.9) |  |
| 70–79 | 91 ( 27.0) | 93 ( 27.6) |  | 44 (26.0) | 49 ( 29.0) |  |
| ≥80 | 173 ( 51.3) | 174 ( 51.6) |  | 87 (51.5) | 87 ( 51.5) |  |
| BMI (%) |  |  | 0.213 |  |  | 0.178 |
| <18.5 | 14 ( 4.2) | 26 ( 7.7) |  | 5 ( 3.0) | 11 ( 6.5) |  |
| 18.5–24.9 | 199 ( 59.1) | 214 ( 63.5) |  | 104 (61.5) | 104 ( 61.5) |  |
| 25–29.9 | 92 ( 27.3) | 74 ( 22.0) |  | 47 (27.8) | 41 ( 24.3) |  |
| ≥30 | 32 ( 9.5) | 23 ( 6.8) |  | 13 ( 7.7) | 13 ( 7.7) |  |
| Diabetes (%) | 147 ( 43.6) | 147 ( 43.6) | 0 | 75 (44.4) | 75 ( 44.4) | <0.001 |
| Hypertension (%) | 242 ( 71.8) | 230 ( 68.2) | 0.078 | 124 (73.4) | 110 ( 65.1) | 0.18 |
| Coronary artery disease (%) | 247 ( 73.3) | 243 ( 72.1) | 0.027 | 127 (75.1) | 120 ( 71.0) | 0.093 |
| Previous heart failure (%) | 127 ( 37.7) | 120 ( 35.6) | 0.043 | 69 (40.8) | 68 ( 40.2) | 0.012 |
| Atrial fibrillation (%) | 132 ( 39.2) | 132 ( 39.2) | 0 | 67 (39.6) | 69 ( 40.8) | 0.024 |
| Previous renal dysfunction (%) | 76 ( 22.6) | 67 ( 19.9) | 0.065 | 42 (24.9) | 33 ( 19.5) | 0.128 |
| Cerebral infarction (%) | 82 ( 24.3) | 79 ( 23.4) | 0.021 | 40 (23.7) | 37 ( 21.9) | 0.042 |
| Cancer (%) | 34 ( 10.1) | 39 ( 11.6) | 0.048 | 11 ( 6.5) | 22 ( 13.0) | 0.221 |
| Cirrhosis (%) | 6 ( 1.8) | 4 ( 1.2) | 0.049 | 2 ( 1.2) | 1 ( 0.6) | 0.063 |
| NYHA classification (%) |  |  | 0.054 |  |  | 0.2 |
| II | 93 ( 27.6) | 85 ( 25.2) |  | 48 (28.4) | 35 ( 20.7) |  |
| III | 155 ( 46.0) | 161 ( 47.8) |  | 80 (47.3) | 82 ( 48.5) |  |
| IV | 89 ( 26.4) | 91 ( 27.0) |  | 41 (24.3) | 52 ( 30.8) |  |
| Paroxysmal nocturnal dyspnea (%) | 62 ( 18.4) | 49 ( 14.5) | 0.104 | 32 (18.9) | 24 ( 14.2) | 0.128 |
| Orthopnoea (%) | 66 ( 19.6) | 61 ( 18.1) | 0.038 | 29 (17.2) | 35 ( 20.7) | 0.091 |
| Heart rate (beats/min) | 82 (70, 99) | 83 (73, 100) | 0.044 | 83 (70, 99) | 83 (72, 100) | 0.003 |
| Systolic blood pressure (mmHg) | 128 (113, 150) | 128 (112, 148) | 0.033 | 130 (114, 151) | 127 (112, 143) | 0.131 |
| Diastolic blood pressure (mmHg) | 70 (60, 82) | 69 (59, 79) | 0.106 | 71 (62, 85) | 70 (60, 80) | 0.134 |
| Rales (>1/2 lung fields) (%) | 127 ( 37.7) | 131 ( 38.9) | 0.024 | 57 (33.7) | 73 ( 43.2) | 0.196 |
| Jugular venous distension (%) | 65 ( 19.3) | 54 ( 16.0) | 0.086 | 30 (17.8) | 34 ( 20.1) | 0.06 |
| Peripheral edema (%) | 218 ( 64.7) | 226 ( 67.1) | 0.05 | 113 (66.9) | 117 ( 69.2) | 0.051 |
| LVEF (%) | 55(45, 60) | 55(45, 60) | 0.008 | 54 (44, 60) | 55 (43, 59) | 0.029 |
| B-type natriuretic peptide (pg/ml) | 874(358, 1760) | 817(384, 2148) | 0.11 | 845 (353, 1884) | 743 (322, 1532) | 0.08 |
| Troponin I(ng/ml) | 0.06 (0.04, 0.10) | 0.06 (0.04, 0.10) | 0.049 | 0.06 (0.04, 0.11) | 0.06 (0.04, 0.1) | 0.123 |
| Hemoglobin (g/L) | 120.9 (24) | 106.2 (23.6) | 0.614 | 115.28 (26.20) | 111.96 (23.14) | 0.135 |
| C-reactive protein (mg/L) | 9.00 (4.16, 26.27) | 13.08 (4.29, 40.29) | 0.147 | 8.71 (4.28, 25) | 8.71 (3.7, 25) | 0.018 |
| Alanine aminotransferase (IU/L) | 16.5 (11.1, 28.2) | 14.7(9.8, 24.1) | 0.153 | 17.4 (10.9, 30.6) | 14.8 (10.4, 24) | 0.201 |
| Total bilirubin (μmol/L) | 13.6(9.6, 20.0) | 12.7 (8.7, 18.9) | 0.065 | 13.7 (9.6, 19.3) | 12.9 (9, 19.2) | 0.086 |
| Blood urea nitrogen (mmol/L) | 8.6 (6.3, 12.4) | 8.7 (6.2, 13.4) | 0.029 | 8.2 (5.9, 12.5) | 8.2 (6.2, 11.7) | 0.094 |
| Creatinine (μmol/L) | 98.7 (75.5, 128.6) | 98.4 (71.5, 144.0) | 0.175 | 96.8 (72.6, 131.2) | 93.4 (73.8, 133.8) | 0.014 |
| Serum albumin (g/L) | 35.2 (34.0, 36.2) | 31.7 (29.2, 33.3) | 0.823 | 34.4 (33.4, 37.9) | 33.3 (32.1, 38) | 0.103 |
| Sodium (mmol/L) | 138.9 (136.0, 141.7) | 138.4 (135.6, 141.6) | 0.041 | 138.9 (136, 141.4) | 138.5 (135.7, 141.7) | 0.1 |
| Potassium (mmol/L) | 4.24 (3.85, 4.62) | 4.10 (3.74, 4.59) | 0.114 | 4.22 (3.86, 4.61) | 4.12 (3.79, 4.59) | 0.084 |
| Uric acid (μmol/L) | 405.1 (312.9, 531.1) | 376.0 (275.0, 503.0) | 0.2 | 409.9 (315, 537.8) | 379.1 (275, 484) | 0.215 |
| Glucose (mmol/L) | 7.19 (5.85, 9.81) | 7.06 (5.67, 9.80) | 0.093 | 7.54 (5.98, 10.6) | 6.9 (5.59, 9.44) | 0.186 |
| Aldosterone antagonists (%) | 249 ( 73.9) | 254 ( 75.4) | 0.034 | 131 (77.5) | 126 ( 74.6) | 0.069 |
| Loop diuretic (%) | 303 ( 89.9) | 321 ( 95.3) | 0.205 | 154 (91.1) | 162 ( 95.9) | 0.193 |
| ACE-Is/ARBs (%) | 139 ( 41.2) | 123 ( 36.5) | 0.098 | 74 (43.8) | 56 ( 33.1) | 0.22 |
| Beta-blockers (%) | 235 ( 69.7) | 238 ( 70.6) | 0.019 | 116 (68.6) | 115 ( 68.0) | 0.013 |
| Anticoagulants (%) | 95 ( 28.2) | 93 ( 27.6) | 0.013 | 49 (29.0) | 43 ( 25.4) | 0.08 |
| Aspirin (%) | 145 ( 43.0) | 150 ( 44.5) | 0.03 | 82 (48.5) | 78 ( 46.2) | 0.047 |
| Vasopressor (%) | 32 ( 9.5) | 26 ( 7.7) | 0.064 | 15 ( 8.9) | 17 ( 10.1) | 0.04 |
| rh-BNP (%) | 0.02 (0.13) | 0.01 (0.11) | 0.049 | 6 ( 3.6) | 6 ( 3.6) | <0.001 |
| NPPV (%) | 0.03 (0.17) | 0.05 (0.21) | 0.092 | 36 (21.3) | 36 ( 21.3) | <0.001 |

Matching strategy 1: Propensity-Score Matching covariates were sex, age, coronary artery disease, heart rate, systolic blood pressure, creatinine, serum albumin, vasopressors, rh-BNP and NPPV. The treatment group were matched in a 1:1 ratio to the control group based on the propensity score without a caliper width.

Matching strategy 2: Propensity-Score Matching covariates were sex, age, coronary artery disease, heart rate, systolic blood pressure, creatinine, serum albumin, vasopressors, rh-BNP and NPPV. The treatment group were matched in a 1:1 ratio to the control group based on the propensity score with a standard caliper width of 0.2.

Data are presented as frequencies (percentages) or mean (SD) or median (IQR)

Abbreviations:

BMI, body mass index (weight in kilograms divided by the square of height in meters)

NYHA, New York Heart Association

LVEF, left ventricular ejection fraction

ACE-Is/ARBs, angiotensin-converting enzyme inhibitors/angiotensin receptor blockers

rh-BNP, recombinant human brain natriuretic peptide

NPPV, non-invasive positive pressure ventilation

Table S8 Sub-group analysis

|  | No albumin | Albumin | HR (95%) | P |
| --- | --- | --- | --- | --- |
| All patients | 701 | 337 | 1.43(1.158-1.765) | 0 |
| Sex |  |  |  |  |
| Male | 342 (48.8) | 154 (45.7) | 1.412(1.04-1.917) | 0.027 |
| Female | 359 (51.2) | 183 (54.3) | 1.531(1.141-2.053) | 0.004 |
| Age |  |  |  |  |
| <60 | 94 (13.4) | 29 ( 8.6) | 2.033(1.039-3.98) | 0.038 |
| ≥60 | 607 (86.6) | 308 (91.4) | 1.379(1.105-1.722 | 0.004 |
| Coronary artery disease |  |  |  |  |
| No | 239 (34.1) | 94 (27.9) | 1.095(0.728-1.645) | 0.664 |
| Yes | 462 (65.9) | 243 (72.1) | 1.603(1.248-2.058) | 0 |
| Diabetes |  |  |  |  |
| No | 409 (58.3) | 190 (56.4) | 1.435(1.075-1.915) | 0.014 |
| Yes | 292 (41.7) | 147 (43.6) | 1.406(1.032-1.917) | 0.031 |
| Hypertension |  |  |  |  |
| No | 213 (30.4) | 107 (31.8) | 1.614(1.075-2.423) | 0.021 |
| Yes | 488 (69.6) | 230 (68.2) | 1.367(1.069-1.749) | 0.012 |
| Previous renal dysfunction |  |  |  |  |
| No | 560 (79.9) | 270 (80.1) | 1.49(1.183-1.878) | 0 |
| Yes | 141 (20.1) | 67 (19.9) | 1.228(0.732-2.058) | 0.435 |
| Systolic blood pressure |  |  |  |  |
| <90 mmHg | 20 ( 2.9) | 9 ( 2.7) | 1.337(0.4-4.464) | 0.637 |
| ≥90 mmHg | 681 (97.1) | 328 (97.3) | 1.435(1.159-1.777) | 0.001 |
| LVEF |  |  |  |  |
| <40% | 148 (21.1) | 67 (19.9) | 1.315(0.815-2.12) | 0.261 |
| 41-49% | 121 (17.3) | 53 (15.7) | 1.295(0.783-2.141) | 0.314 |
| ≥50% | 432 (61.6) | 217 (64.4) | 1.575(1.203-2.062) | 0 |
| NYHA |  |  |  |  |
| II | 183 (26.1) | 85 (25.2) | 1.118(0.718-1.741) | 0.621 |
| III | 333 (47.5) | 161 (47.8) | 1.484(1.098-2.006) | 0.01 |
| IV | 185 (26.4) | 91 (27.0) | 1.688(1.135-2.511) | 0.009 |


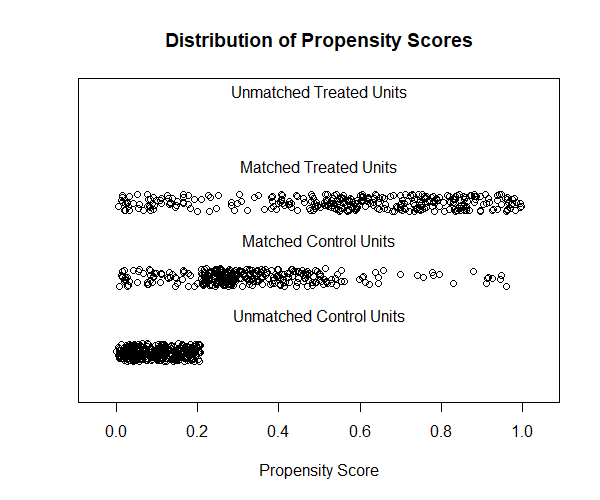


Figure S1. Distribution of the estimated propensity score for receiving albumin, among patients who did and did not actually receive the treatment


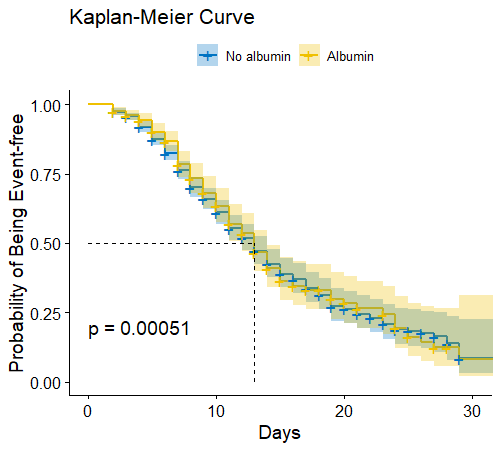


Figure S2. Freedom from Composite End Point with inverse probability weighting according to the propensity score.

The shaded areas represent pointwise 95% confidence intervals.
